# Widespread legacy effects on net primary productivity across western US drylands

**DOI:** 10.1007/s00442-025-05785-2

**Published:** 2025-08-22

**Authors:** Isabella R. Goodman, Andrew J. Felton

**Affiliations:** 1 Department of Land Resources and Environmental Sciences, Montana State University, Bozeman, MT 59717 USA; 2 Yale School of the Environment, Yale University, New Haven, CT 06511 USA

**Keywords:** Drylands, NPP, Legacy effects, Climate change, Climate extremes

## Abstract

Despite the water-limited nature of dryland ecosystems, interannual variability in precipitation (PPT) fails to explain a significant fraction of interannual variability in net primary productivity (NPP). One hypothesis states that these weak temporal NPP-PPT associations arise from the lagged effects of previous-year conditions, denoted as “legacy effects,” which may amplify or constrain NPP in subsequent years. Although evidence suggests the existence of legacy effects in many ecosystem types, their generality in drylands remains unclear. We used long-term (35-year) remotely sensed estimates of NPP, climate, and a vegetation structure across the western United States to quantify the sign, magnitude, and drivers of legacy effects, defined as the lagged effects of previous-year weather and NPP anomalies on current-year NPP. Legacy effects exert a widespread effect on interannual variability in NPP across drylands spanning annual and perennial grasslands to hot and cold deserts. Previous-year NPP anomalies were the strongest predictor of current-year NPP anomalies, both across the entire time series and during specific extreme-to-average year transitions. The association between previous- and current-year NPP anomalies was consistently positive, indicating that a productive previous year will tend to result in a productive current year, and vice versa, even after accounting for the effect of current-year PPT. The strength of legacy effects increased slightly with increasing mean annual PPT and decreased slightly with an increase in the average fraction of herbaceous NPP. We conclude that legacy effects consistently effect current-year NPP in drylands and that consideration of these effects can improve predictions of temporal variation in dryland NPP.

**Supplementary Information:**

The online version contains supplementary material available at 10.1007/s00442-025-05785-2.

## Introduction

Although ecosystem functioning in drylands is primarily regulated by water availability, the association between precipitation (PPT) and net primary productivity (NPP) is far weaker through time than across space (Huxman et al. 2004; Sala et al. 2012). At regional scales, spatial gradients in average annual PPT are strongly, positively correlated with spatial gradients in average annual NPP (Felton et al. 2021a, b; Maurer et al. 2020; Sala et al. 1988). In contrast, interannual variability in annual PPT is often a much weaker predictor of interannual variability in annual NPP in drylands (Gherardi and Sala 2019; Knapp and Smith 2001). This spatial–temporal contrast in PPT–NPP relationships (Felton et al. 2021a, b; Sala et al. 2012) illustrates a persistent limitation in our ability to predict the effects of climate change on dryland functioning; we can anticipate the effects of changes in climatic averages, but not increasing climatic variability.

The limited ability of current-year PPT to predict current-year NPP may reflect that temporal variability in dryland ecosystem functioning is shaped not only by immediate responses to current environmental conditions (e.g., current-year PPT) but also by a history of prior conditions (Delgado-Balbuena et al. 2019) that generate hysteresis in ecosystem functioning. Growing evidence suggests that previous-year climatic conditions (e.g., drought) can produce lagged effects on ecosystems that influence NPP in subsequent years (Anderegg et al. 2015; de Vries et al. 2012; Griffin-Nolan et al. 2018; Sala et al. 2012). Such lagged effects on NPP can be driven by a range of mechanisms, including alterations to plant hydraulics (Anderegg et al. 2015), plant community composition (Delgado-Balbuena et al. 2019), or meristem densities (Griffin-Nolan et al. 2018; Reichmann et al. 2013). Changes in plant community structure, whether via plant mortality or changes in species composition, following climate extremes or other disturbances may thus dictate the following year’s production; if previous-year conditions increase mortality, the following year will likely have low production. These lingering alterations to ecosystems, often following disturbances, are *collectively* denoted as “legacy effects” and may manifest as lags on ecosystem processes, such as NPP. While previous research has observed that legacy effects can influence temporal variation in NPP (Delgado-Balbuena et al. 2019; Griffin-Nolan et al. 2018; Sala et al. 2012), the sign, magnitude, and generality of these effects remains unclear.

**Fig. 1:**
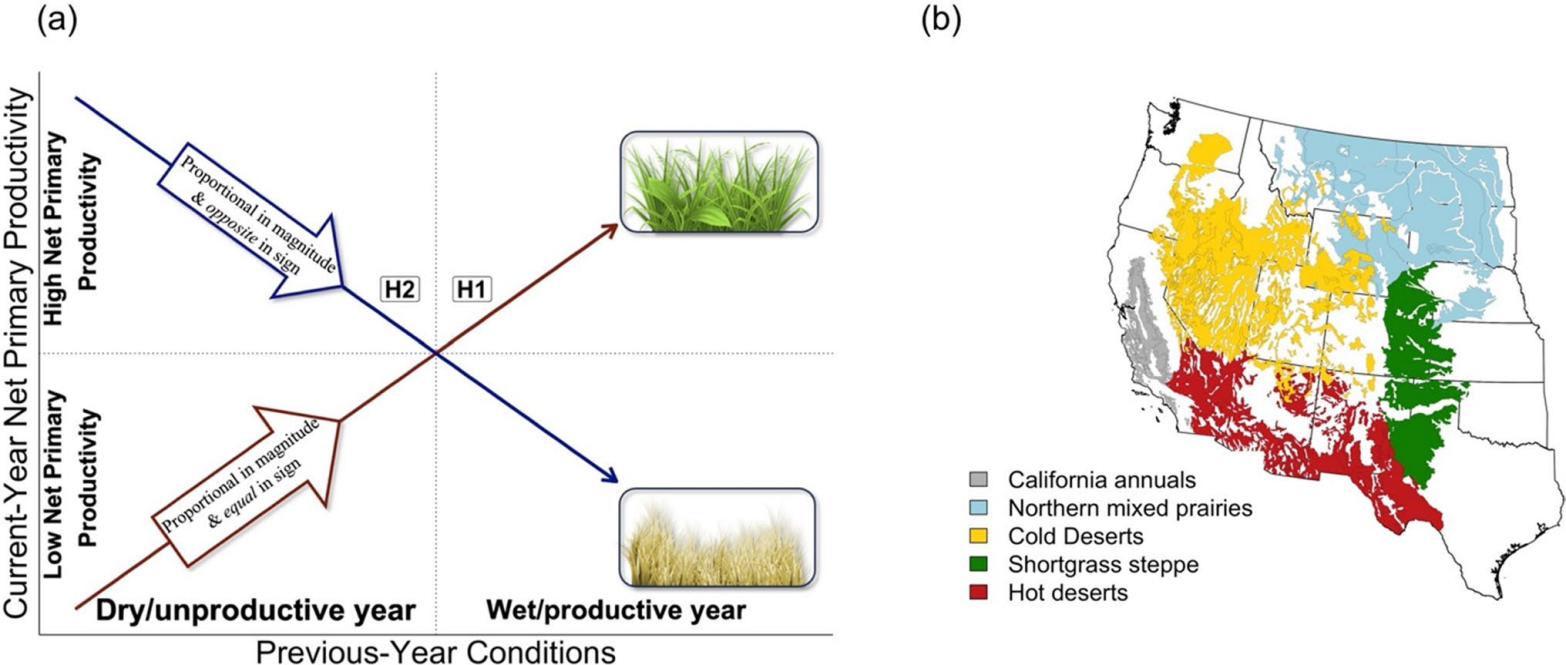
**a** Predictions of the two key hypotheses for legacy effects of previous-year conditions on current-year net primary productivity. Hypothesis 1 (H1), the **linear-positive hypothesis**, predicts that legacy effects are proportional in magnitude and *equal* in sign to previous-year conditions; a productive or wet previous year will lead to the following year being productive as well. Hypothesis 2 (H2), the **linear-negative hypothesis,** predicts that legacy effects are proportional in magnitude yet *opposite* in sign to previous-year conditions; an unproductive or dry year will lead to a productive succeeding year (Sala et al. 2012). **b** Spatial distribution of the five dryland ecoregion types (Kuchler 1964; Allred et al 2021; Felton et al. 2021a, b) used in our analysis across the western United States

Sala et al. 2012 proposed two key hypotheses concerning NPP legacies: the linear-positive hypotheses and the linear-negative hypothesis (Fig. 1a). The linear-positive hypothesis (Fig. 1a, H1) states that legacy effects are proportional in magnitude and *equal* in sign to previous-year conditions, be they abiotic (PPT, temperature) or biotic (NPP). Both structural and biogeochemical mechanisms could drive this in drylands (Sala et al. 2012). For example, an exceptionally dry year may increase plant mortality and decrease plant densities that in turn constrains NPP in the following years, even if PPT is above average. This “vegetation constraint” has been experimentally shown in both North and South American drylands (Reichmann et al. 2013; Yahdjian and Sala 2006). High temperatures may also cause additional heat stress and water stress that is not fully encoded in PPT (Dannenberg et al. 2022; Diffenbaugh et al. 2015). Similarly, an exceptionally wet year may increase plant densities that could enhance exploitation of limiting resources and thus enhance NPP in the following year, even if PPT is below average (Sala et al. 2012). Biogeochemically, a highly productive year could also increase soil nutrient availability in subsequent years via enhanced litter inputs (Delgado-Balbuena et al. 2019; Hassan et al. 2021), thus decreasing resource (nutrient) co-limitation (e.g., soil nitrogen) and enhancing NPP in the following year (Hooper & Johnson 1999). Low litter inputs following an unproductive year may also decrease soil inorganic nitrogen and subsequently constrain NPP (Xu et al. 2013). A key prediction of the linear-positive hypothesis is that an unproductive year will result in the following year also being unproductive, and vice versa (Fig. 1a, H1).

Alternatively, the linear-negative hypothesis states that legacy effects are proportional in magnitude but *opposite* in sign to previous-year conditions (Fig. 1a, H2) (Sala et al. 2012). A proposed mechanism for this hypothesis concerns trophic dynamics. For example, following an unproductive year, above- and belowground herbivory pressures may be decreased because of a lack of plant tissues to support herbivory, resulting in higher-than-expected NPP in the subsequent year. Following a productive year, high amounts of plant tissues may result in elevated herbivory pressures that constrain plant growth, resulting in lower-than-expected NPP (Bakker et al. 2006; Detling 1988). A secondary mechanism results from cost–benefit relationships. Following a wet year, plants may deploy too many leaves and roots, resulting in high respiratory or water use costs. Warm temperatures in spring may again cause plants to deploy too many leaves and roots early that cannot be sustained later in the summer when the plant becomes water stressed (Marsh and Zhang 2022). If PPT in the following year is low or even average, plants may not obtain enough carbon or water to support these new structures, resulting in a skewed energy and water balance and, thus, a negative legacy (Sala et al. 2012; Zhao et al. 2022). Biogeochemically, N mineralization rates also tend to be less impacted by drought, resulting in pulses of limiting nutrients following drought which could potentially enhance NPP, particularly if moisture is not limiting in the following year (Hofer et al. 2017). A key prediction of the linear-negative hypothesis is that an unproductive year will result in the following year being productive, and vice versa (Fig. 1a, H2).

Previous research suggests that legacy effects on NPP exist across a diversity of terrestrial ecosystems (Griffin-Nolan et al. 2018; Kannenberg et al. 2020; Sala et al. 2012; Wu et al. 2018). On one hand, there is widespread evidence for the linear-positive hypothesis; a synthesis of long-term field studies in drylands (deserts to grasslands) found that legacy effects on aboveground NPP were proportional to the magnitude of previous-year conditions and equal in sign (Sala et al. 2012). Similarly, another study in temperate northern hemisphere grasslands found that plant growth was reduced for up to two years following extreme drought (Wu et al. 2018). On the other hand, there is evidence for the linear-negative hypothesis; one study in the US Great Plains found that one year of extreme drought led to increased aboveground NPP in the following year (Griffin-Nolan et al. 2018), a finding that was mirrored across Inner Mongolian grasslands (Sun et al. 2022). Thus, evidence exists to support predictions of both the linear-negative and linear-positive hypotheses (Fig. 1a).

One common limitation of prior research on legacy effects is the relatively small spatial scale and limited number of sites analyzed (Griffin-Nolan et al. 2018; Sala et al. 2012). A more general understanding of legacy effects would require NPP data with broader spatial and temporal coverage than field studies can provide. Remote sensing estimates of NPP achieve this by providing not only broad spatial coverage of ecosystems and ecoregions but now broad temporal (> 30 years) coverage as well (Robinson et al. 2019). The resulting spatiotemporal coverage of these data introduces new possibilities. For example, while previous synthesis work of field studies suggests that the strength of legacy effects is independent of mean annual PPT, such results were based on a limited number (*n* = 16) of sites for an average of 21 years (Sala et al. 2012). Assessing the generality of previous assessments of legacy effects is now possible with the temporal maturation of remote sensing estimates of NPP, and recent innovations in remote sensing estimates of NPP, such as the partitioning of NPP into different functional groups, have focused on western US drylands specifically (Robinson et al. 2019). Together, the temporal maturation and innovation in remote sensing data have opened new opportunities to assess legacy effects across a wider range of ecosystems, regions, and time periods.

The goal of this study was to assess the generality of legacy effects as a driver of interannual variation in NPP across dryland ecosystems. We specifically asked three questions: (1) Do legacy effects, as proxied by previous-year anomalies in weather or NPP, improve predictions of interannual variation in dryland NPP, relative to models that just use current-year weather anomalies? (2) What is the sign and magnitude of legacy effects and how do these effects vary across spatial gradients of climate and vegetation structure? (3) How do legacy effects modify the sensitivity of current-year NPP to current-year PPT? To this end, we conducted a targeted analysis on the role of legacy effects across five western United States (US) dryland ecoregions (Fig. 1b), specifically by building competing spatiotemporal models of remotely sensed NPP, weather, and vegetation structure that incorporated differing previous-year biotic and climatic “lag” variables as a proxy for legacy effects. We report evidence for widespread linear-positive legacy effects across western US drylands (Fig. 1a, H2), the strength of which increases as average water availability increases and decreases as the abundance of herbaceous vegetation NPP increases.

## Material and methods

### Study region

This study focused on drylands of the western United States (US), which span a diversity of ecosystem types including deserts, shrublands, and grasslands (Hoover et al. 2020), all of which are limited by water availability (Bradford et al. 2020; Liu et al. 2023; Sala et al. 1988). Consistent with previous work in this region (Felton et al. 2021a, b), we grouped western US drylands into five ecoregions: California annual grasslands, northern mixed prairies, cold deserts, shortgrass steppe, and hot deserts. The boundaries for these ecoregions were delineated based on the Kuchler (1964) map of potential natural vegetation that was further scrutinized using remote sensing derived partitioned vegetation cover data (Allred et al. 2021; Felton et al. 2021a, b; Robinson et al. 2019). Each ecoregion varied in vegetation composition consistent with expectations while also varying in mean annual PPT, mean NPP, and mean temperature (Table S1), as reported previously (Felton et al. 2021a, b). The distinct climatic regimes and vegetation types among these ecoregions further provided a natural experiment to assess spatial variability in legacy effects across different dryland types.

### Core datasets

#### Precipitation and temperature

Annual precipitation (PPT) data in units of mm were obtained from the DAYMET V4 Daily Surface Weather and Climatological Summaries. These data provide long-term continuous daily gridded estimates of weather (Thornton et al. 2020) and were obtained and processed using Google Earth Engine. The PPT data were aggregated from a native pixel resolution of 1000 m to 1500 m and summed to represent annual water-year PPT, defined as the sum of October 1 to September 30 PPT (Felton et al. 2021a, b). Temperature data were also obtained from the DAYMET V4 Daily Surface Weather and Climatological Summaries and aggregated from a native pixel resolution of 1000 m to 1500 m. Daily minimum and maximum temperature data were averaged at the daily and then annual water-year scale to represent annual temperature.

#### Net primary productivity

Net primary productivity (NPP) data in units of grams of carbon per square meter (g C m^−2^) were obtained from the Rangeland Analysis Platform (Robinson et al. 2019). These data estimate the net amount of carbon fixed into plant biomass over a calendar year. We used the partitioned NPP data product provided by the Rangeland Analysis Platform, which includes, for each pixel, annual NPP estimates (here from 1986 to 2021) for annual forbs and grasses, perennial forbs and grasses, shrubs, and trees (Table S1) (Robinson et al. 2019). For most of our analyses, all NPP subgroups were summed into one total NPP value for each pixel-year combination. However, to calculate the fraction of herbaceous NPP (percentage of NPP from herbaceous vegetation) for later use as a covariate, we summed together only NPP from annual forbs and grass and perennial forbs and grasses. Nevertheless, herbaceous plants dominated NPP across this dataset; the average percent of total NPP from herbaceous vegetation ranged from 70% in the hot deserts to 96% in the northern mixed prairies (**Table S2**). We used a multi-step process to filter out pixels irrelevant to the study (e.g., irrigated croplands). We first used the USGS National Land Cover Database to filter pixels to that fell within herbaceous/grassland and shrub/scrub landcover types (Dewitz 2021) (**Methods S1**). The data were then aggregated from a native pixel resolution of 30 m (Robinson et al. 2019) to a pixel resolution of 1500 m and were additionally filtered in a multi-step procedure to remove outliers/non-rain fed areas to ensure we accurately captured the dynamics of a natural (e.g., non-irrigated) dryland ecosystems (see Methods S1 for detailed information) (Felton et al. 2021a, b). Overall, this process resulted in 32,815,160 pixel-year combinations.

### Core analyses

#### Selection of lag variable to quantify legacy effects.

To assess the role of previous-year conditions – namely, weather or production – on annual NPP, we first needed to select a previous-year lag variable to represent such effects. In all models, we represented annual values for a given variable as annual *anomalies*, quantified as the annual deviation from the 35-year mean for a given pixel. We considered multiple lag variables including previous-year NPP, PPT, and temperature and found that previous-year NPP anomalies were a far better predictor of current-year NPP anomalies than either previous-year PPT or temperature anomalies (Analysis S1 for detailed information). Thus, previous-year NPP anomalies served as our legacy effect variable; these legacy effects derived from rates of prior-year productivity are henceforth referred to as *NPP legacy effects*.

To further reduce the effects of spatial autocorrelation on the uncertainty surrounding coefficient estimates from our models, a spatial block bootstrapping process was implemented, following the approach of Felton et al. 2021a, b. This process was implemented for each model described hereafter. In short, pixels were stratified based on (1) the ecoregion in which they were embedded and (2) whether they fell below or above the mean annual PPT (MAP) for that ecoregion. 0.1% of pixels were then randomly selected within this stratification. This procedure was repeated 1000 times with replacement, and, for each iteration, we extracted multiple model metrics including the R-squared, Akaike information criterion (AIC), and slope coefficients, thus generating distributions of each metric.

*Q1: Does the inclusion of legacy effects improve predictions of temporal variation in annual NPP in drylands?*

To answer question 1, we determined if and to what extent previous-year NPP anomalies, i.e., NPP legacy effects, was a relevant predictor of current-year NPP anomalies after accounting for current-year PPT anomalies, a commonly used predictor of temporal variation in NPP in drylands (Felton et al. 2021a, b; Gherardi and Sala 2019; Knapp et al. 2017; Reichmann et al. 2013). To this end, we created two models: one with current-year PPT anomalies as the sole predictor variable, and one that included both current-year PPT and previous-year NPP anomalies as main effects:

1 $${NPPdev}_{x,t}=\alpha +\sigma {PPT}_{x,t}+{\varepsilon }_{x,t},$$

2 $${NPPdev}_{x,t}=\alpha +\sigma {PPT}_{x,t}+\tau {lagNPP}_{x,t-1}+{\varepsilon }_{x,t},$$

where ${NPPdev}_{x,t}$ denotes current-year NPP anomalies from the long-term mean at location *x* and time *t*, $\alpha$ denotes the intercept term, $\sigma$ denotes a temporal slope that provides the relationship between current-year NPP anomalies and current-year PPT anomalies, and $\tau$ denotes a temporal slope that provides the relationship between current-year NPP anomalies and previous-year NPP anomalies at time* t* -1.

We chose this approach to analyze the effect of including previous-year NPP rather than regressing against the residuals of Eq. 1 (Sala et al. 2012), or in other words the anomalies from the predictions of Eq. 1., because residual regression risks generating bias from correlated variables (Freckleton 2002; Garcia-Berthou 2001). Building a multiple linear regression including both current-year PPT and previous-year NPP anomalies thus avoided this potential bias. To compare the performance of the two models, both the *R*^2^ values and AIC scores were extracted from each model and the differences were calculated (for each iteration). We determined whether these differences were statistically “significant” by estimating their mean and 99% confidence intervals; if they failed to overlap with zero, we deemed the differences between the two models statistically different from zero. We further assessed the degree of existing temporal autocorrelation, both in the model without (Eq. 1) and with (Eq. 2) a lagged variable as a predictor, using Durbin Watson Tests. In both cases, we found evidence for weak positive autocorrelation in the models, the magnitude of which did not appear to vary in subsequent, more complex models (Figs. S3, S4, see supporting information for detailed discussion).

*Q2: What is the sign and magnitude of legacy effects and how do they vary across gradients of mean water availability and vegetation structure?*

To answer question 2, we first mapped out previous-year NPP anomaly coefficients, τ, from running 937,576 pixel-specific regression models using Eq. 2. This allowed us to isolate and map the effect (coefficient) of previous-year NPP anomalies after accounting for current-year PPT anomalies and begin to understand the general trends in the sign and magnitude of NPP legacy effects across the entire region.

To determine if and how NPP legacy effects vary across spatial gradients of mean water availability and vegetation structure, we built two additional models. Equation 3 determines how NPP legacy effects vary along a gradient of mean annual PPT (MAP), while Eq. 4 determines how NPP legacy effects vary along gradients of herbaceous NPP. Herbaceous NPP represents the average amount (%) of total NPP that comes from herbaceous vegetation.

3 $${NPPdev}_{x,t}=\alpha +\beta {Eco}_{x}+\gamma {Eco}_{x}{PPT}_{x,t}+\upomega {Eco}_{x}{MAP}_{x}+\uptheta {Eco}_{x}{lagNPP}_{x,t-1}+\delta {Eco}_{x}{lagNPP}_{x,t-1}{MAP}_{x}+{\varepsilon }_{x,t},$$

4 $$NPPdev_{x,t} = \alpha + \beta Eco_{x} + \gamma Eco_{x} PPT_{x,t} + \omega Eco_{x} HERB_{x} + \theta Eco_{x} lagNPP_{x,t-1} + \delta Eco_{x} lagNPP_{x,t-1} HERB_{x}+{\varepsilon }_{x,t},$$

where $\beta$ denotes the main effect of ecoregion, $\gamma$ denotes how the effect of current-year PPT varies with ecoregion, $\upomega$ denotes how the effect of MAP (Eq. 3) or herbaceous NPP (Eq. 4) varies with ecoregion, and $\delta$ denotes how the effect of the interaction between previous-year NPP and MAP (Eq. 3) or herbaceous NPP (Eq. 4) varies by ecoregion.

To further understand NPP legacy effects within the context of extreme years, we focused on transitions from “extreme” to “normal” year transitions within each ecoregion. We specifically focused on NPP anomalies the year following 1) the *driest* and *wettest* years and 2) the *least* and *most* productive years. We assessed the NPP anomaly in the year following those extremes only for pixels that met our criteria. Pixels were only included if the succeeding year’s PPT was within 1 standard deviation of the mean annual PPT for that pixel. This was done to avoid potential effects of multi-year droughts or wet periods (Felton et al. 2021a, b), which is beyond the scope of this study. We then looked at correlations (using Spearman’s r) between NPP anomalies in the wettest/driest or least/most product year and NPP anomalies in the succeeding “normal” year.

*Q3: How do legacy effects influence the sensitivity of current-year NPP to current-year PPT?*

To answer question 3, we created a model with the interaction between current-year NPP anomaly, current-year PPT anomaly, and ecoregion:

5 $${NPPdev}_{x,t}=\alpha +\beta {Eco}_{x}+\gamma {Eco}_{x}{PPT}_{x,t}+\uptheta {Eco}_{x}{lagNPP}_{x,t-1}+\delta {Eco}_{x}{lagNPP}_{x,t-1}{PPT}_{x,t}+{\varepsilon }_{x,t},$$

where $\uptheta$ denotes how the effect of previous-year NPP anomaly varies by ecoregion and $\delta$ denotes the interaction between previous-year NPP and current-year PPT, providing how the effect of current-year PPT on current-year NPP varies according to previous-year NPP.

## Results

*Q1: Does the inclusion of legacy effects in models improve predictions of interannual variation in dryland NPP?*

Yes. Predictions of current-year NPP anomalies improved significantly when previous-year NPP anomalies was added to the model including only current-year PPT anomalies. For example, R^2^ values from the bootstrapped model iterations improved from a mean of 0.24 in the bivariate model including only current-year PPT anomalies (Eq. 1) to a mean of 0.44 when previous-year NPP was added, thus increasing R^2^ values by 83.4% on average (**Fig. S2b, Table S3**). Shifts in AIC values were of a far smaller magnitude but supported the R^2^ results; on average, AIC values were reduced by just 3.1% after incorporating previous-year NPP anomalies (**Fig. S2a, Table S3**). 99% confidence intervals for both R^2^ and AIC differences did not overlap with zero, supporting the result that previous-year NPP anomalies improved model predictions of current-year NPP anomalies, even after incorporating the effects of current-year PPT anomalies. Results were qualitatively the same when all variables were standardized to have a mean of 0 and standard deviation of 1 (**Fig. S8**).

*Q2: What is the sign and magnitude of legacy effects and how do they vary across gradients of mean water availability and vegetation structure?*

**Fig. 2:**
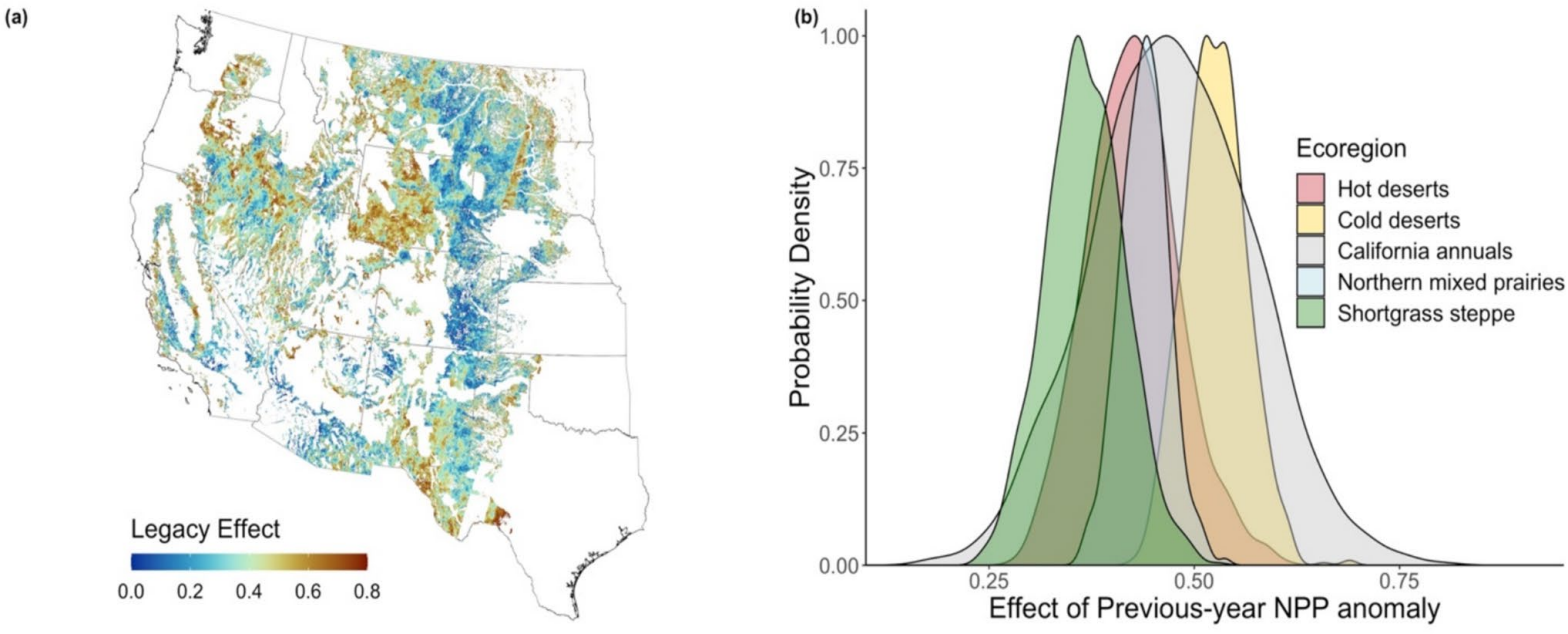
**a** Spatial distribution of previous-year NPP anomaly coefficient ($\tau$) from pixel-specific regressions of Eq. 2. Pixels falling above the 97.5th percentile or below the 2.5th percentile of the $\tau$ distribution were removed to better show spatial variation. **b** Probability density functions (scaled to 1) of the effect (slope) of previous-year NPP anomalies on current-year NPP anomalies for each ecoregion from Eq. 5

Previous-year NPP anomaly coefficients from both pixel-specific regressions and the bootstrapping process of Eq. 2 were consistently positive in sign across the study region (Fig. 2**, S3**). In other words, the relationship between previous-year NPP anomalies and current-year NPP anomalies was consistently positive, indicating that current-year NPP anomalies tended to be *equal* in sign to previous-year anomalies. The effect of previous-year NPP varied by ecoregion (Fig. 2b**, S3**); mean coefficient values (from Eq. 5) representing the effect of previous-year NPP ranged from 0.37 in the shortgrass steppe to 0.52 in the cold deserts, indicating that current-year NPP anomalies were *not* equal in magnitude to previous-year anomalies. Nevertheless, 99% confidence intervals of the mean coefficient from each ecoregion showed no overlap across ecoregions (**Table S4**).

**Fig. 3:**
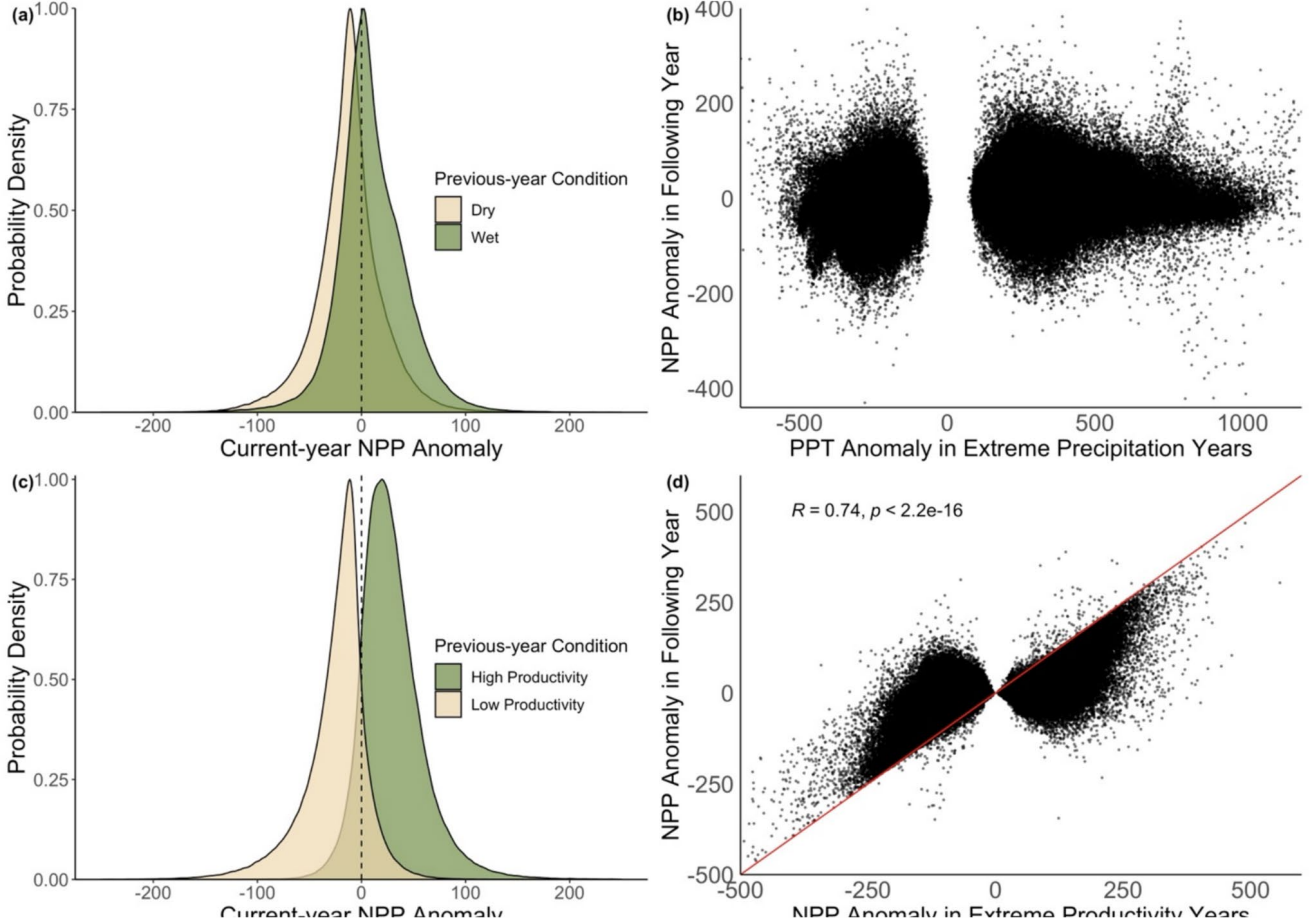
**a** Probability density functions (scaled to 1) of NPP anomalies following extremely wet or dry years and **b** bivariate relationships between NPP anomalies in extreme PPT years and the following “normal” years. **c** Probability density functions (scaled to 1) of NPP anomalies following extremely productive or unproductive years and **d** bivariate relationships between NPP anomalies in extreme NPP years and the following years. Red line in panel **d** represents the 1:1 line. Years were defined as extreme if they had the highest or lowest pixel-specific anomaly from the 35-year mean PPT or NPP, and the following year was “normal” if it was within one standard deviation of the pixel-specific mean annual PPT

NPP anomalies following both PPT, temperature, and productivity extremes further indicated linear-positive legacy effects. However, NPP extremes had a stronger effect on succeeding year NPP anomalies than PPT or temperature extremes (Fig. 3**, Fig. S4**), as shown by both probability density functions of NPP anomalies (Fig. 3a, c**, Fig. S4a**) and bivariate relationships between current and following year NPP anomalies (Fig. 3b, d**, Fig. S4b**). This result was confirmed by Spearman’s correlation coefficients; the coefficient representing the Spearman’s correlation between NPP anomalies in extreme productivity years and NPP anomalies in the succeeding year was 0.74 (*p*-value < 2.2e-16), while the coefficients representing the Spearman’s correlations between extreme PPT years and NPP anomalies in the following year and extreme temperature years and NPP anomalies in the following years were 0.36 and 0.41, respectively, though both were still statistically significant (*p*-value < 2.2e-16). The magnitude of anomalies also varied by whether the extreme year was wet versus dry. For example, Spearman’s coefficient values were 0.04 (*p*-value < 2.2e-16) for the correlation between NPP anomalies in the wettest year and NPP anomalies in the succeeding year, yet were 0.24 (*p*-value < 2.2e-16) in the driest years. These patterns held true when we used a more restrictive criteria to define “normal” years by only including observations where the following year PPT was within 0.25 standard anomalies of the mean.

**Fig. 4:**
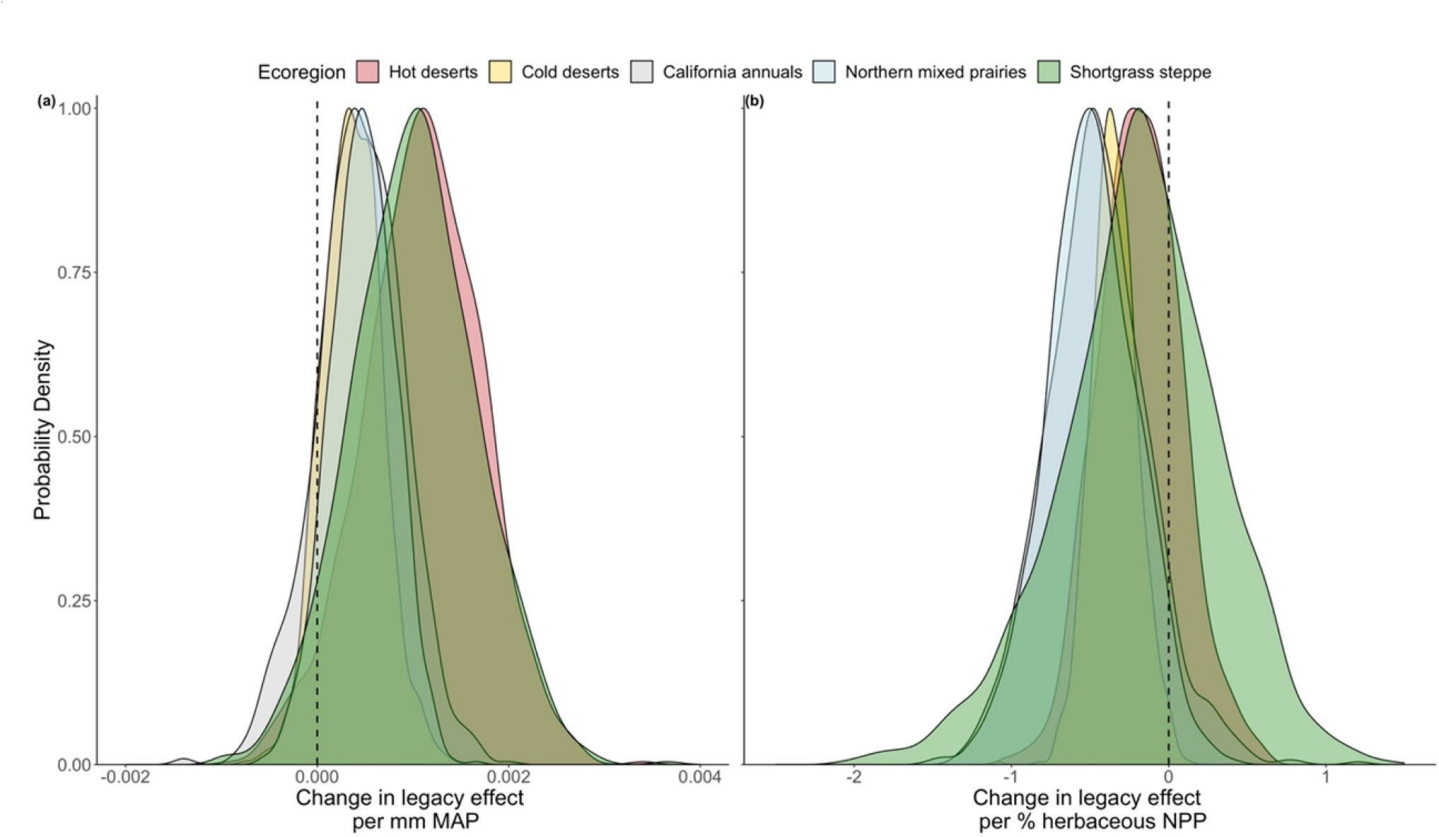
Probability density functions (scaled to 1) of **a** coefficients from the interaction between ecoregion, MAP, and previous-year NPP (Eq. 3) and **b** coefficients from the interaction between ecoregion, the fraction of herbaceous NPP, and previous-year NPP (Eq. 4). The control of previous-year NPP on current-year NPP increases with mean annual precipitation and decreases as the fraction of herbaceous NPP increases

We observed that the strength of NPP legacy effects tended to increase as mean annual PPT increased. This trend was consistent across all five ecoregions and thus did not depend strongly on ecoregion; probability density functions for the interaction term coefficients had considerable overlap (Fig. 4a). Mean coefficients ranged from 0.00036 in the cold deserts to 0.0011 in the hot deserts (**Table S5**). Although there appears to be overlap of the change in legacy effect per mm mean annual PPT (Fig. 4a) across the ecoregions, 99% confidence intervals of the mean coefficient value suggest that the ecoregions are statistically different, except for California annuals/northern mixed prairies which were statistically similar (99% confidence intervals overlapped) (**Table S5**). The effect of mean annual PPT on the strength of NPP legacy effects in the hot deserts was 101.4% greater than the effect in the cold deserts. Although the relative increase is high, the absolute difference between ecoregions is small, as shown by the large overlap between ecoregions (Fig. 4a).

Alternatively, coefficients representing the change in NPP legacy effects due to herbaceous NPP, from Eq. 4, were primarily negative (Fig. 4b). This indicates that NPP legacy effects tend to decrease as the average percentage of NPP from herbaceous vegetation increases. When analyzed across ecoregions this relationship varied only slightly. California annual grasslands had the lowest interaction term coefficient of -0.45 and the hot deserts had the highest of -0.18 (**Table S6**). This suggests that the California annuals ecoregion sees the greatest decrease in NPP legacy effects per 1% increase in herbaceous NPP and the hot deserts ecoregion sees the least decrease. 99% confidence intervals show no overlap across ecoregions (**Table S6**), except for between the shortgrass steppe and the hot deserts. This indicates statistically significant differences in the magnitude of the effect across most ecoregions, despite clear overlap between ecoregions in probability density functions and small absolute differences among ecoregions (Fig. 4b).

*Q3: How do legacy effects influence the sensitivity of current-year NPP to current-year PPT?*

**Fig. 5:**
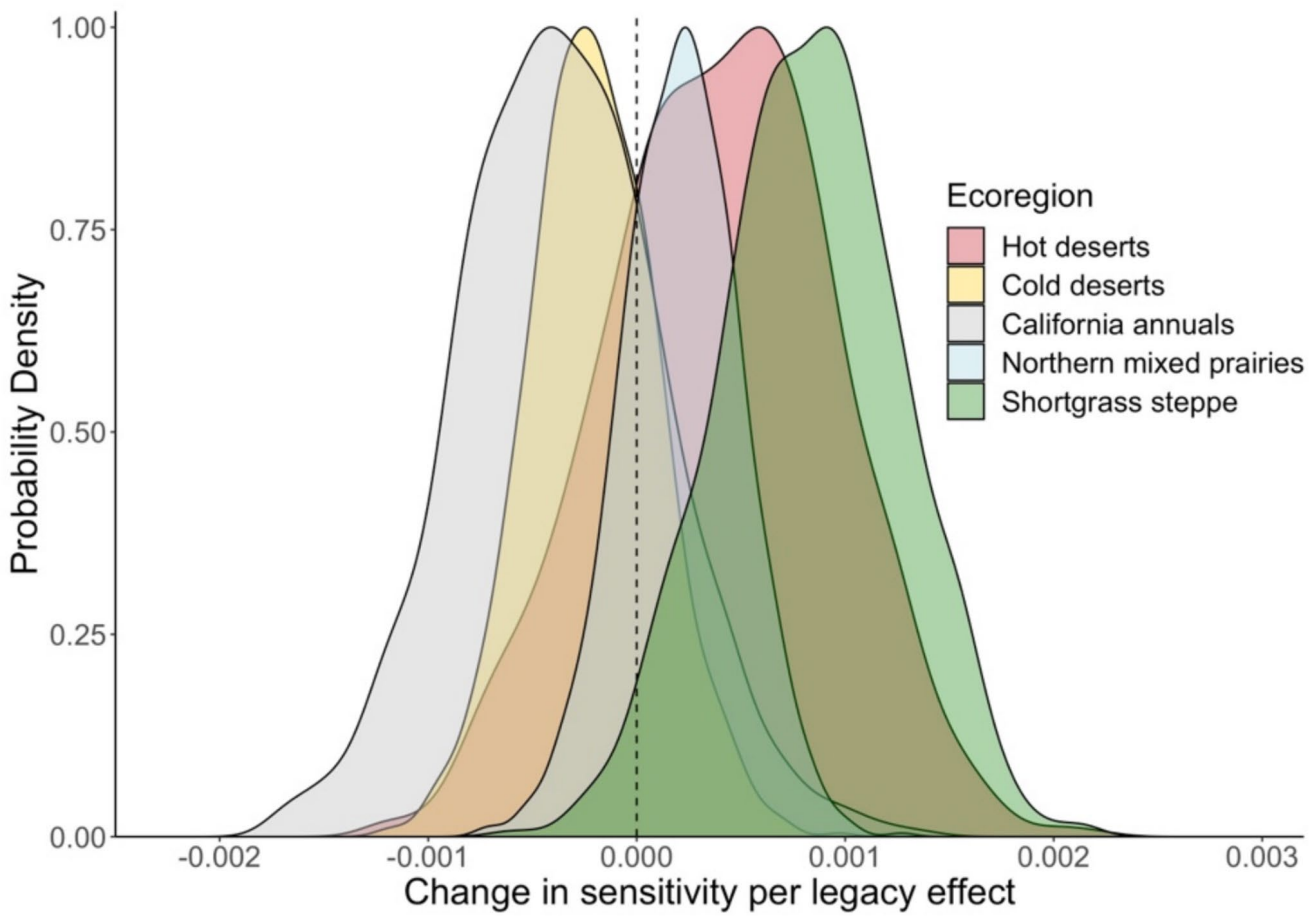
Probability density functions (scaled to 1) of coefficients from the interaction between ecoregion, current-year PPT, and previous-year NPP, showing the change in sensitivity of current-year NPP to current-year PPT

Inconsistently and weakly. Coefficients from the interaction term between current-year PPT anomaly and previous-year NPP anomaly suggested a small influence of previous-year NPP on the sensitivity of current-year NPP to current-year PPT. This effect was inconsistent within and across ecoregions (Fig. 5). For example, in the shortgrass steppe, northern mixed prairies, and hot deserts, the interaction was positive. As previous-year NPP anomalies became more positive, so too did the slope of current-year PPT anomalies, indicating that a productive year will *increase* the effect of current-year PPT on current-year NPP in the succeeding year, and vice versa. This relationship was strongest in the shortgrass steppe (0.00083). By contrast, in the cold deserts, and the California annuals, this interaction was negative; as previous-year NPP anomalies became more positive, the slope of current-year PPT anomalies became more *negative,* indicating that a productive year will tend to *decrease* the effect of current-year PPT on current-year NPP, and vice versa*.* The negative relationship was strongest in the California annuals (– 0.00036). Although mean coefficients differed between the ecoregions, likely due to the large sample size, probability density functions showed clear overlap both among ecoregions and with zero (Fig. 5), thus indicating a relatively weak and inconsistent effect that was again qualitatively the same when standardizing variables in our models (**Fig. S6).**

## Discussion

Our most important finding is evidence for a widespread influence of legacy effects on interannual variation in NPP across western US drylands. In our study this influence was realized specifically through NPP legacy effects, defined as the temporal slope between current-year and previous-year NPP anomalies, which were equal in sign (though not in magnitude) to previous-year conditions (Fig. 1H 1**, **Fig. 2**, **Fig. 3**, S3**). In other words, a productive year will tend to be followed by another productive year, and vice versa. These effects were apparent after accounting for the influence of current-year PPT anomalies (**Fig. S2**) and were apparent in years following extreme positive and negative NPP anomalies (Fig. 3c, d). Thus, previous-year NPP exerts a general effect on current-year NPP across drylands, despite interannual variability in NPP being relatively high in these ecosystems. By contrast, evidence for hypothesized PPT legacies (Delgado-Balbuena et al. 2019; Reichmann et al. 2013; Sala et al. 2012) was weak and was most evident following extreme dry PPT anomalies (Fig. 3a, b). Given the apparent ubiquitous effect of previous-year NPP on current-year NPP across these drylands (Fig. 2**, S3**), the inclusion of these legacy (or lagged) effects significantly increased the amount of interannual variation explained in NPP (**Fig. S2b, Table S3**). Our results further suggest that the strength and influence of these NPP legacy effects can be modified by spatial variability in both mean water availability, via changes in mean annual PPT, and vegetation structure, through changes in the amount of herbaceous vegetation. Coupling our large-scale results with similar results from small-scale observational (Sala et al. 2012) and experimental (Broderick et al. 2022; Reichmann et al. 2013) field studies suggests that these NPP legacy effects exert a widespread influence on temporal variability in dryland NPP.

### Legacy effects

After considering multiple lag variables as proxies of NPP legacy effects, including PPT and temperature, previous-year NPP anomalies emerged as the strongest predictor of current-year NPP anomalies across all five dryland ecoregions (**Table S3, **Fig. S1**, Analysis S1**). Our findings are consistent with previous findings from research conducted in the field setting. For instance, Sala (2012) also found that previous-year aboveground NPP explained a larger amount of variation in current-year aboveground NPP than previous-year PPT, and in our case we find very limited support for PPT legacies across remotely sensed time series (**Table S3, **Fig. S1). Rather, we found some support for legacies following extreme drought (Fig. 3b), which often results in large reductions in NPP (Cao et al. 2022; Lei et al. 2020). That previous-year PPT would not strongly explain current-year NPP should not be surprising given the often weak association between current-year PPT and NPP (Felton et al. 2021a, b; Knapp et al. 2017); indeed, this weak temporal association was a key motivation for this work. And while it is perhaps not surprising that NPP would be correlated with a lagged version of itself, we suggest that our results offer more than a simple demonstration of temporal autocorrelation of time series data (Bence 1995). We propose that previous-year NPP emerged as a superior predictor of current-year NPP because of its ability to capture the lagged effects of other dryland NPP drivers beyond weather, such as fire (Brown and Collins 2023), herbivory (Davis et al. 2015), and soil nutrients (Burke et al. 1997; Carbonell-Silletta et al., n.d.), all of which can have a direct effect on NPP (Field et al. 1995; McGuire et al. 1992; Melillo et al. 1993).

The positive relationship between previous- and current-year NPP anomalies provides support for the hypothesis of linear-positive NPP legacy effects in drylands (Fig. 1a, H1**, (**Sala et al. 2012**)** and also likely has a simple underlying mechanism in drylands. We suggest that the dominant mechanism, and most parsimonious explanation, underpinning this relationship in drylands is alterations to vegetation structure and specifically vegetation density. The amount of primary productivity in a given year places a fundamental constraint on primary productivity in the following year, likely due to changes in stem (or belowground bud) and root densities and leaf area (Reichmann et al. 2013; Yahdjian and Sala 2006) needed for resource acquisition, especially in water-limited biomes. Indeed, Reichmann et al. (2013) found that nearly 40% of the variation of legacy effects of aboveground NPP in a Southwestern US dryland were explained by changes in tiller (stem) density from the previous year. This result from a field experiment connects to our remote sensing result that NPP anomalies were the strongest predictor of current-year NPP anomalies across the entire time series (**Table S3, **Fig. S1**, Analysis S1**) *and* following extreme years (Fig. 3), broadly supporting the role of structural mechanisms in driving NPP legacy effects in drylands. This potentially indicates that climatic extremes do not necessarily produce NPP legacy effects in drylands and that they would be most likely to do so if 1) an extreme drought occurred and, importantly, 2) the drought altered vegetation structure, density, and or/production potential.

### Controls of spatial variation in legacy effects

We found that the effect of previous-year NPP on current-year NPP increased slightly from drier to wetter sites (Fig. 4a). This result contrasts with Sala (2012), who found no clear effect of mean annual PPT on the magnitude of NPP legacy effects, though we suspect that this finding is due to the limited number (*n* = 16) of dryland sites analyzed, in contrast to the 937,576 pixels analyzed here. Further analysis of interannual variation in our data (expressed as the coefficient of variation of NPP) showed that temporal variation in NPP decreased as mean annual PPT increased across all ecoregions (**Fig. S5**). At a surface level this is perhaps not surprising and underpins the association between NPP legacy effects and mean annual PPT. At a deeper level, we suggest this has a mechanistic basis. We hypothesize that NPP legacy effects increase from dry to wet sites because of shifting controls of water availability to resource co-limitation by nitrogen or light (Bharath Iyengar 2020; Eskelinen and Harrison 2015; Hooper and Johnson 1999; Huxman et al. 2004), which is influenced by spatiotemporal variation in water availability (Eskelinen and Harrison 2015; Felton et al. 2021a, b, p. 2; Huxman et al. 2004). Observed reductions in the sensitivity of annual NPP to PPT as mean annual PPT increases provide some support for this hypothesis (Felton et al. 2021a, b; Huxman et al. 2004; Maurer et al. 2020). At historically wetter sites in drylands, the presumed limiting resources are soil nutrients, such as nitrogen, which we posit is less temporally variable than soil moisture, the likely limiting resource in drier locations. The result is a reduction in the temporal variability of NPP at wetter locations and thus an apparent enhanced effect of previous-year NPP on current-year NPP (Fig. 4a).

In contrast to mean annual PPT, we found that as the percentage of herbaceous (e.g., grasses and forbs) NPP increased, the strength of NPP legacy effects decreased. The magnitude of this effect varied across ecoregions and was the least pronounced in the cold deserts and most pronounced in the shortgrass steppe (Fig. 4b). These findings are partially consistent with the results from a study in southwest China, which found that legacy effects in trees and shrubs were stronger and persisted longer than those in grasses (Ma et al. 2023; Wu et al. 2018). Prior cross-biome analyses suggest that grasslands have the highest aboveground NPP variability (Knapp and Smith 2001), and thus, we hypothesize that drylands with a high percent of herbaceous NPP exhibit greater interannual variability of NPP. Indeed, additional analysis on the interannual variability of NPP across a gradient of herbaceous NPP confirmed that as the percent of NPP from herbaceous species increases, the variation in NPP also increases (**Fig. S6**). Increased interannual NPP variability in herbaceously dominated areas thus leads to a weaker effect of previous-year NPP on current-year NPP and likely enhances the influence of current-year PPT on NPP (Felton, Shriver, et al., 202). As a result, average moisture availability and herbaceous NPP exert opposing effects on the degree to which previous-year NPP influences current-year NPP in drylands. We suspect that these results explain the ecoregion level differences in legacy effects (Fig. 2a).

Variation in vegetation structure in turn changes an ecoregion’s temporal variability in NPP (Gherardi and Sala 2019; Knapp and Smith 2001). Variation in rooting strategies of vegetation may especially act an important structural mechanism via its control on temporal NPP dynamics. For instance, deep rooted woody plants (Dodd and Lauenroth 1997; Sala et al. 1989), such as the shrubs co-dominant across much of the cold deserts, may be less sensitive to previous-year conditions given their overall low sensitivity to soil moisture variability (Tredennick et al. 2018), generating lower production variability and by extension higher apparent NPP legacies. This is likely because deeper rooted plants tend to access deeper and more stable soil water pools (Lauenroth et al. 2014). Shallow-rooted herbaceous vegetation acts as a natural contrast to shrubs, given their high sensitivity to soil moisture and precipitation variability (Terry et al. 2024) and high production variability (Knapp and Smith 2001). Herbaceous vegetation also has a propensity to quickly recovery after disturbance (Ruppert et al. 2015), such as extreme drought (Hoover et al. 2014), thus weakening the control of previous-year production on current-year production. Consequently, areas characterized by woody vegetation, like the cold deserts, may be expected to have stronger apparent NPP legacy effects than herbaceously dominated sites like the shortgrass steppe – where the NPP legacy effects were the weakest (Fig. 2a).

### Alterations of the sensitivity of current-year NPP to current-year PPT due to legacy effects

Last, we found that previous-year NPP weakly and inconsistently alters the sensitivity of current-year NPP to current-year PPT (Fig. 5). Given the bounds of these effects span negative to positive, the two ecoregions at those bounds provide a framework for exploring potential mechanisms. At one bound, the shortgrass steppe had the strongest *positive* interaction, suggesting that a high previous-year NPP will result in current-year PPT having an increased effect on current-year NPP. We suspect that this positive interaction could be due to alterations in bud bank and plant stem densities (Felton et al. 2021a, b; Yahdjian and Sala 2006). An increase of plants each year means a greater potential of responses and therefore less of a vegetation constraint. Thus, changes in previous-year plant densities are likely to affect the responsiveness of current-year NPP to current-year PPT (Reichmann and Sala 2014) by simply enhancing the potential magnitude of response to PPT, particularly in systems already highly sensitive to current-year PPT. At the other bound, the California annuals had the strongest *negative* interaction. As previous-year NPP increased, the sensitivity of current-year NPP to current-year PPT decreased and thus a highly productive year will likely result in low sensitivity the following year, and vice versa. We suspect that this negative interaction could be due, in part, to litter accumulation of annual plants. High amounts of litter accumulation resulting from productive years may form a layer that subsequently enhances light limitation (Facelli and Pickett 1991; Hou et al. 2019), thereby constraining NPP.

### Limitations

An easy criticism of this work is that we have, in so many words, simply rediscovered temporal autocorrelation in time series data. The basic nature of our analysis – correlating a variable against a lagged version of itself – alludes to this common issue in time series data (Bence 1995; Wolkovich et al. 2014), as it can lead to non-independence in observations, higher variance, altered significance levels, and thus, skewed results. Indeed, we find multiple lines of evidence for weak positive temporal autocorrelation of NPP anomalies, both through formal testing, model coefficients (Fig. 2), and comparisons of specific extreme-to-normal year transitions (Fig. 3), which is consistent with the hypothesis that previous-year primary production should influence, to some degree, current-year primary production (Fig. 1**,** Sala et al. 2012).

Our study and analyses, however, are explicitly focused on understanding legacy effects and thus the role of prior conditions in shaping a process, not unlike prior analyses on this topic (Anderegg et al. 2015; Griffin-Nolan et al. 2018; Reichmann et al. 2013; Sala et al. 2012). We thus chose to do our analysis this way because it encodes the effect of previous-year conditions on current-year NPP and gives us a way to quantify the direction and magnitude of legacy effects. In other words, there is a mechanistic basis and indeed alternative hypotheses (Fig. 1a) motivating our approach for exploring the strength of these lagged effects on current-year NPP in drylands. Thus, while these previous- and current-year NPP associations are in effect an illustration of temporal autocorrelation, and there are more perhaps complex model structures or approaches we – or in the future others – could have pursued, the weakness of this apparent autocorrelation renders us skeptical that we would arrive at a fundamentally different conclusion. Rather, we contend that this autocorrelation encodes important information on the drivers of temporal variation in NPP in drylands and shows support for prior hypotheses and smaller-scale, mechanistic field studies for how the legacies of prior years can influence current-year primary productivity in drylands.

### Implications

We have shown that NPP legacy effects are a widespread driver of temporal variability in NPP – a key carbon cycle process – across a diversity of Western North American dryland ecoregions. Previous-year NPP exerts a consistent effect, at least in the direction of its effect, on current-year NPP in ecosystem types spanning annual to perennial grasslands and hot to cold deserts. Low NPP years appear to constrain production in subsequent years, and high NPP years appear to enhance production in subsequent years. Spatial variation in the magnitude of these NPP legacy effects arose in part because they strengthened slightly as average moisture availability increased across sites and weakened slightly as herbaceous vegetation increased across sites. This suggests that concurrent and future changes to climate and vegetation structure will modify the magnitude of hysteresis in dryland functioning moving forward.

As climate change initiates directional changes in precipitation, we can expect to see gradual changes in NPP trends as well, from either direct increases or decreases in NPP due to changes in PPT, changes in temperature, or changes in species composition driven by water availability. For example, if climate change leads to drier ecosystems, productivity will generally decrease and if climate change leads to wetter ecosystems, productivity will generally increase. Although this is the assumption, the magnitude of this response is also likely dictated by a multitude of other factors such as the length of the drought/deluge, and the time since occurrence, neither of which our model can predict. We also suspect that changes in plant composition will also alter the magnitude of hysteresis. For example annual grass invasions (Bansal and Sheley 2016) will likely reduce the effect of previous-year NPP on current-year NPP, while woody encroachment (Li et al. 2022; Ratajczak et al. 2012) will likely enhance the influence of previous-year NPP on current-year NPP. Thus, although our research provides a framework for understanding what directional changes mean for NPP legacy effects, additional research would be needed to determine how the magnitude of hysteresis changes with multi-year droughts, and to understand how long NPP legacy effects last.

Finally, this research also has implications and applications for management in dryland systems of the western US. The roughly $100 billion beef industry in the US (Reeves et al. 2017) is primarily supported by livestock production in the arid and semi-arid regions of the western US that in turn relies on estimates of forage production. Understanding the potential control of previous-year NPP on current-year NPP can provide valuable insight into what the forage availability may be like in the following year. Additionally, NPP legacy effects may provide insight into ecosystem restoration and sequestration strategies. With restoration, an understanding that efforts to return NPP to its previous state may be delayed following a dry and/or exceptionally unproductive year may provide insight into proper restoration methods. Similarly, in carbon sequestration strategies, understanding NPP legacies and that both previous- and current-year conditions may alter carbon inputs can aid in realistic sequestration estimates and efforts moving forward.

## Supplementary Information

Below is the link to the electronic supplementary material.

Supplementary file1 (PDF 2948 kb)

## Data Availability

Core data and code used for this analysis are publicly available in a GitHub repository: https://github.com/isabellargoodman/Legacy-effect-project.
