# Supplementary material for: Widespread legacy effects on net primary productivity across western US drylands
Source: Oecologia. 2025 Aug 22;207(9):149. doi: 10.1007/s00442-025-05785-2 (PMC12373685; doi:10.1007/s00442-025-05785-2)
Supplement: Supplementary file 1 — Supplementary file1 (PDF 2948 kb) [file 442_2025_5785_MOESM1_ESM.pdf]

Table S1. Table showing ecoregion specific NPP for each plant functional group (annual grasses and forbs, perennial grasses and forbs, shrubs, and trees), total NPP and PPT means 95% confidence intervals. NPP values are in g/m^2.

Ecoregion	Annual grasses and forbs NPP		Perrenial grasses and forms NPP		Shrubs NPP		Trees NPP		Total NPP		PPT	
	Mean	*95% CI*	*Mean*	*95% CI*	*Mean*	*95% CI*	*Mean*	*95% CI*	*Mean*	*95% CI*	*Mean*	*95% CI*
California Annuals	171.9	(171.76,172.08)	57.43	(57.32,57.54)	25.9	(25.01,25.16)	64.3	(64.19,64.59)	323.59	(323.33,323.85)	556.02	(555.42,556.63)
Cold Deserts	16.19	(16.17,16.2)	58.91	(58.88,58.94)	17.61	(17.60,17.62)	10.9	(10.89,10.93)	104.83	(103.99,104.07)	353.75	(353.66,353.85)
Hot Deserts	10.06	(10.04,10.08)	48.95	(48.90,48.98)	17.17	(17.15,17.19	6.4	(6.38,6.42)	82.89	(82.85.82.95)	301.22	(301.11,301.33)
Northern Mixed Prairies	17.03	(17.02.17.04)	164.9	(164.89,194.91)	2.7	(2.69,2.71)	2.42	(4.41,4.43)	190.16	(190.11,190.21)	424.93	(424.83,425.02)
Shortgrass Steppe	17.65	(17.64,17.69)	148.6	(148.56,148.68)	4.89	(4.88,4.90)	2.77	(2.76,2.78)	175.11	(175.11,175.18)	438.47	(438.34,438.60)

Table S2. Ecoregion specific mean proportion of NPP that is from herbaceous plants (Annual and perennials) and 99% confidence intervals.

Ecoregion	fracHERB	
	Mean	*99% CI*
California Annuals	0.7714	(0.7709,0.7721)
Cold Deserts	0.7118	(0.7117,0.7120)
Hot Deserts	0.7008	(0.7006,0.7011)
Northern Mixed Prairies	0.9629	(0.9628,0.9629)
Shortgrass Steppe	0.9575	(0.9573,0.9576)

Table S3. Ecoregion specific coefficient means and 99% confidence intervals of lagNPP coefficient from Eq. 5.

Ecoregion	lagNPP	
	Mean	*99% CI*
California Annuals	0.4736	(0.4657, 0.4815)
Cold Deserts	0.5258	(0.5228, 0.5288)
Hot Deserts	0.4257	(0.4212, 0.4302)
Northern Mixed Prairies	0.4392	(0.4369, 0.4417)
Shortgrass Steppe	0.3671	(0.3632, 0.3709)

Table S4. Ecoregion specific coefficient means and 99% confidence intervals from the spatiotemporal interaction term (lagNPP x MAP) from model 4 Coefficient estimates were obtained from the 1000 iterations of the bootstrapping process.

**Ecoregion**	**lagNPP x MAP**	
	Mean	*99% CI*
California Annuals	0.0004427	(0.0004056,0.0004797)
Cold Deserts	0.0003653	(0.0003403,0.0003904)
Hot Deserts	0.0011082	(0.0010585,0.0011578)
Northern Mixed Prairies	0.0004742	(0.0004471,0.0005013)
Shortgrass Steppe	0.0010247	(0.0009722,0.0010772)

Table S5. Ecoregion specific coefficient means and 99% confidence intervals from the spatiotemporal interaction term (lagNPP x fracHERB) from model 4. Coefficient estimates were obtained from the 1000 iterations of the bootstrapping process.

**Ecoregion**	**fracHERB x lagNPP**	
	Mean	*99% CI*
California Annuals	-0.4521	(-0.4785,-0.4258)
Cold Deserts	-0.3455	(-0.3574,-0.3336)
Hot Deserts	-0.1809	(-0.2023,-0.1595)
Northern Mixed Prairies	-0.4919	(-0.5152,-0.4685)
Shortgrass Steppe	-0.1866	(-0.2279,-0.1452)

Table S6: Ecoregion specific coefficient means and 99% confidence intervals from the spatiotemporal interaction term (lagNPP x currentPPT) from model 5. Coefficient estimates were obtained from the 1000 iterations of the bootstrapping process.

**Ecoregion**	**currentPPT x lagNPP**	
	Mean	*99% CI*
California Annuals	-0.0003619	(-0.0004018,-0.0003221)
Cold Deserts	-0.0002097	(-0.0002351,-0.00018420
Hot Deserts	0.0003952	(0.0003505,0.0004399)
Northern Mixed Prairies	0.0002138	(0.0001906,0.0002371)
Shortgrass Steppe	0.0008303	(0.0007951,0.0008655)

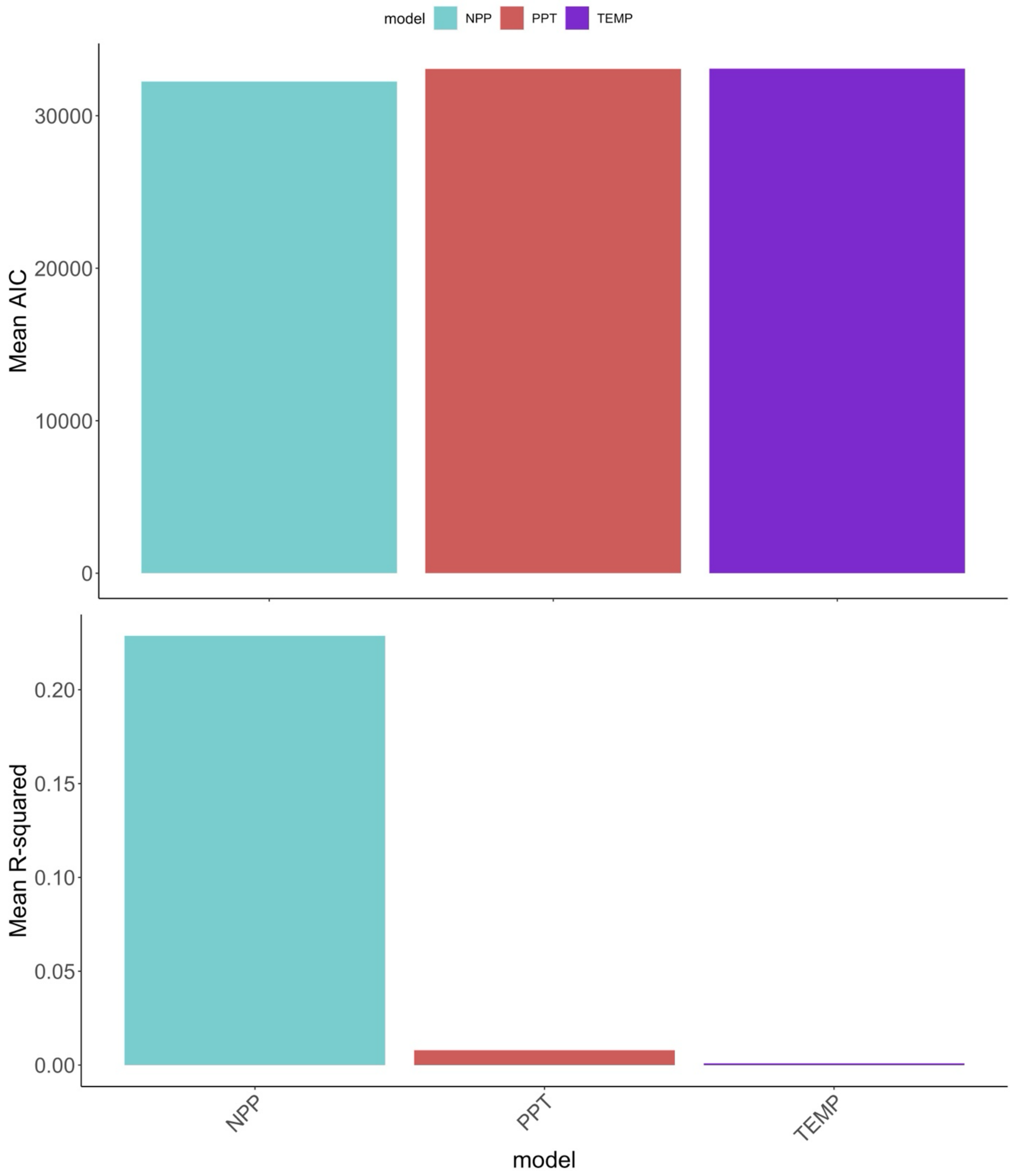

Figure S1. Histogram of R-squared and AIC values for previous year NPP, TEMP, and PPT models for all ecoregions combined.

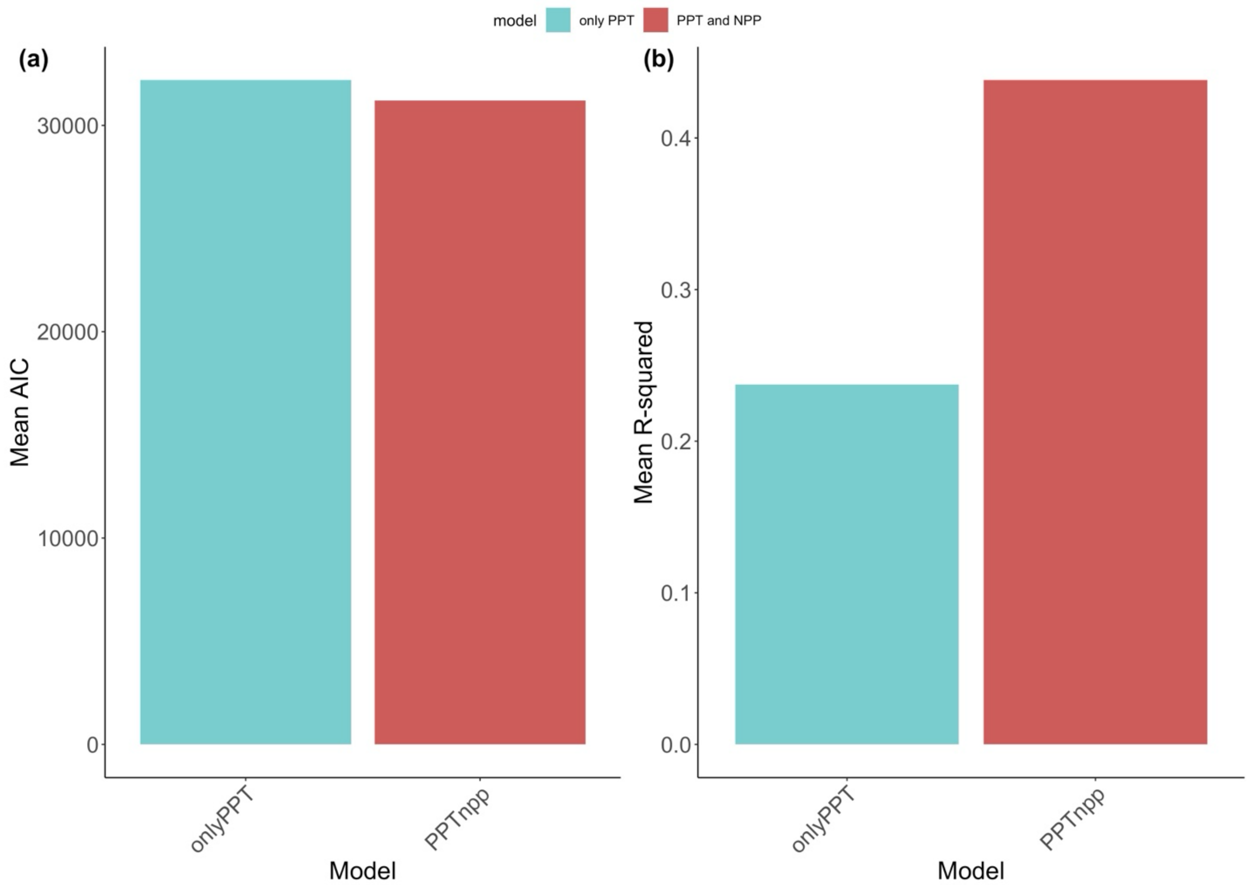

Figure S2. Histogram of **(a)** AIC and **(b)** R-Squared values for models 3 ($NPP_{x,t} = \alpha + \sigma PPT_x + \varepsilon_{x,t}$) (blue) and 4 $NPP_{x,t} = \alpha + \sigma PPT_x + \tau lagNPP_{x,t} + \varepsilon_{x,t}$ (red) for all ecoregions combined.

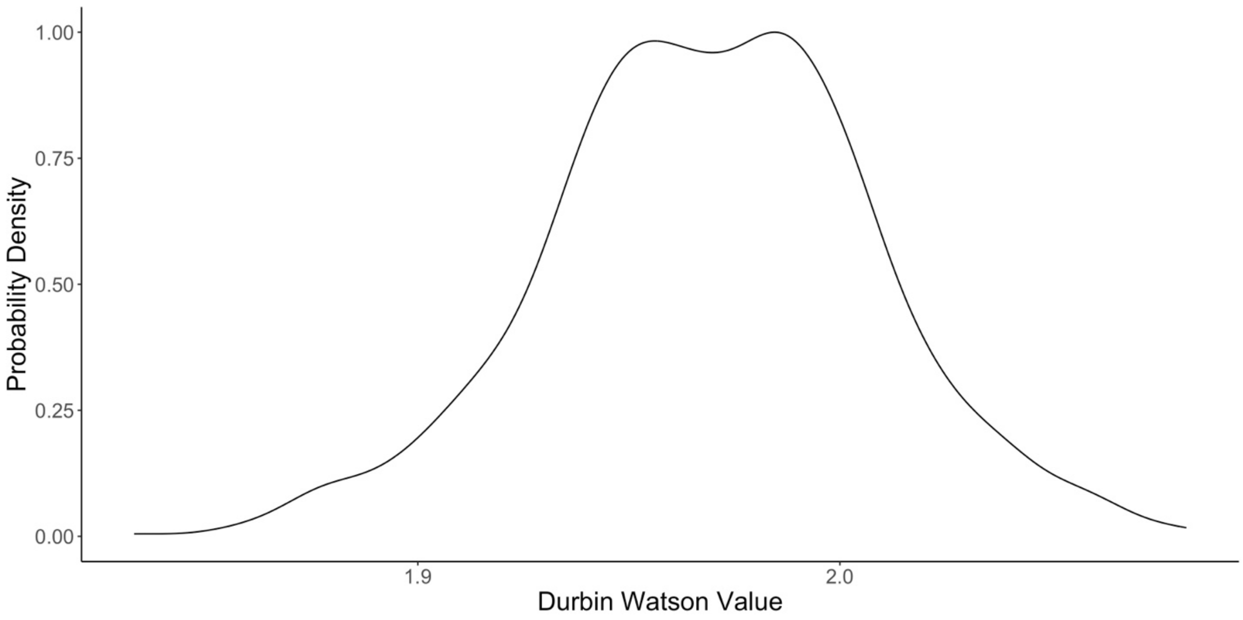

Figure S2. Probability density functions of Durbin Watson DW test statistic values scaled to one from all 1000 iterations of Eq. 1. The mean value of these iterations (X) indicated weak positive autocorrelation.

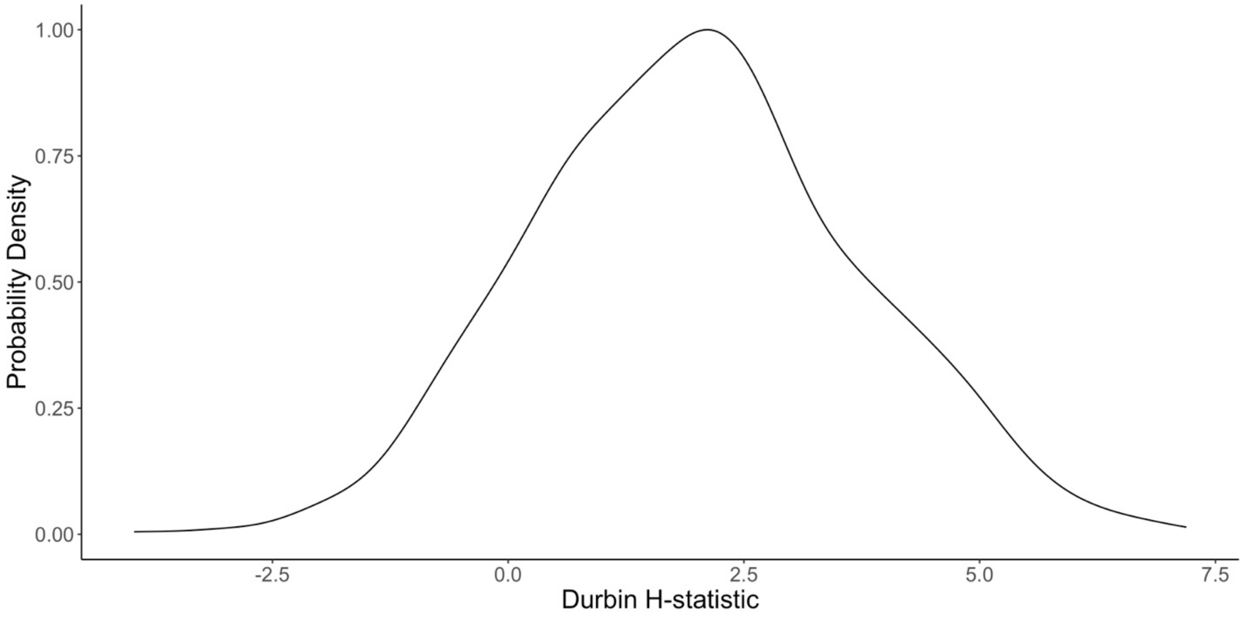

Figure S3. Probability density functions of Durbin Watson H test statistic values scaled to one from all 1000 iterations of Eq. 2. The mean value of these iterations (1.98) indicated weak positive autocorrelation.

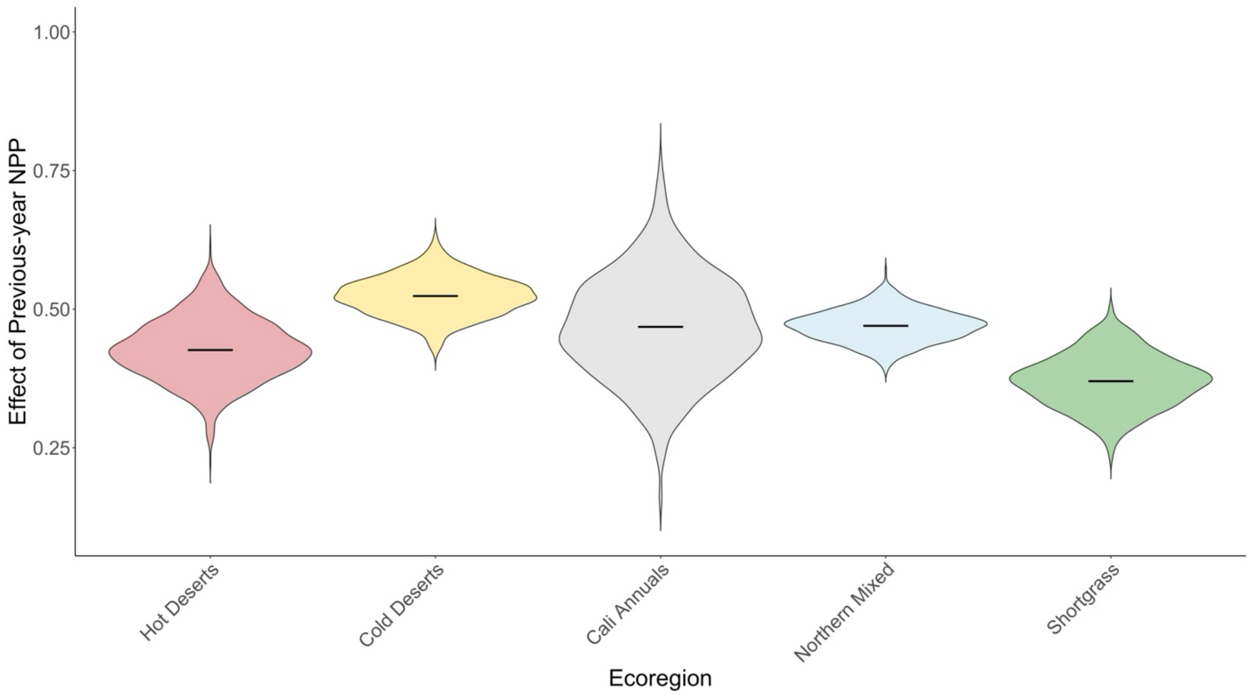

Figure S5. Violin plots of the effect (slope) of previous-year NPP anomalies on current-year NPP anomalies for each ecoregion from Eq. 5.

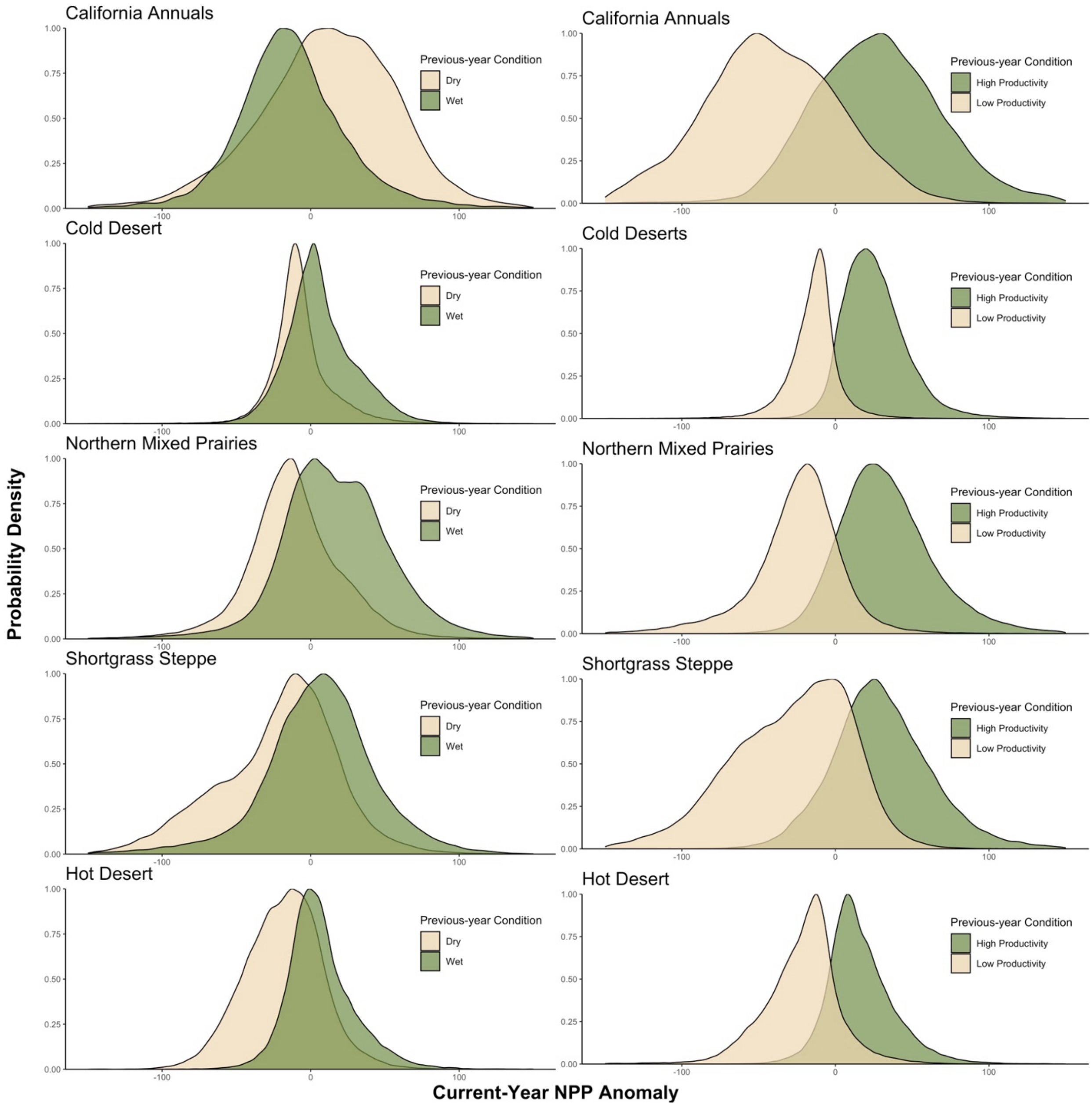

Figure S6. Probability density functions of NPP deviation following exceptionally productive years and exceptionally unproductive years and exceptionally wet and exceptionally dry years across the five dryland ecoregions. Years were quantified as exceptionally productive/unproductive and wet/dry if they had the highest or lowest pixel specific deviation from the mean PPT or NPP and the following year was within one standard deviation of the mean precipitation or productivity for that pixel.

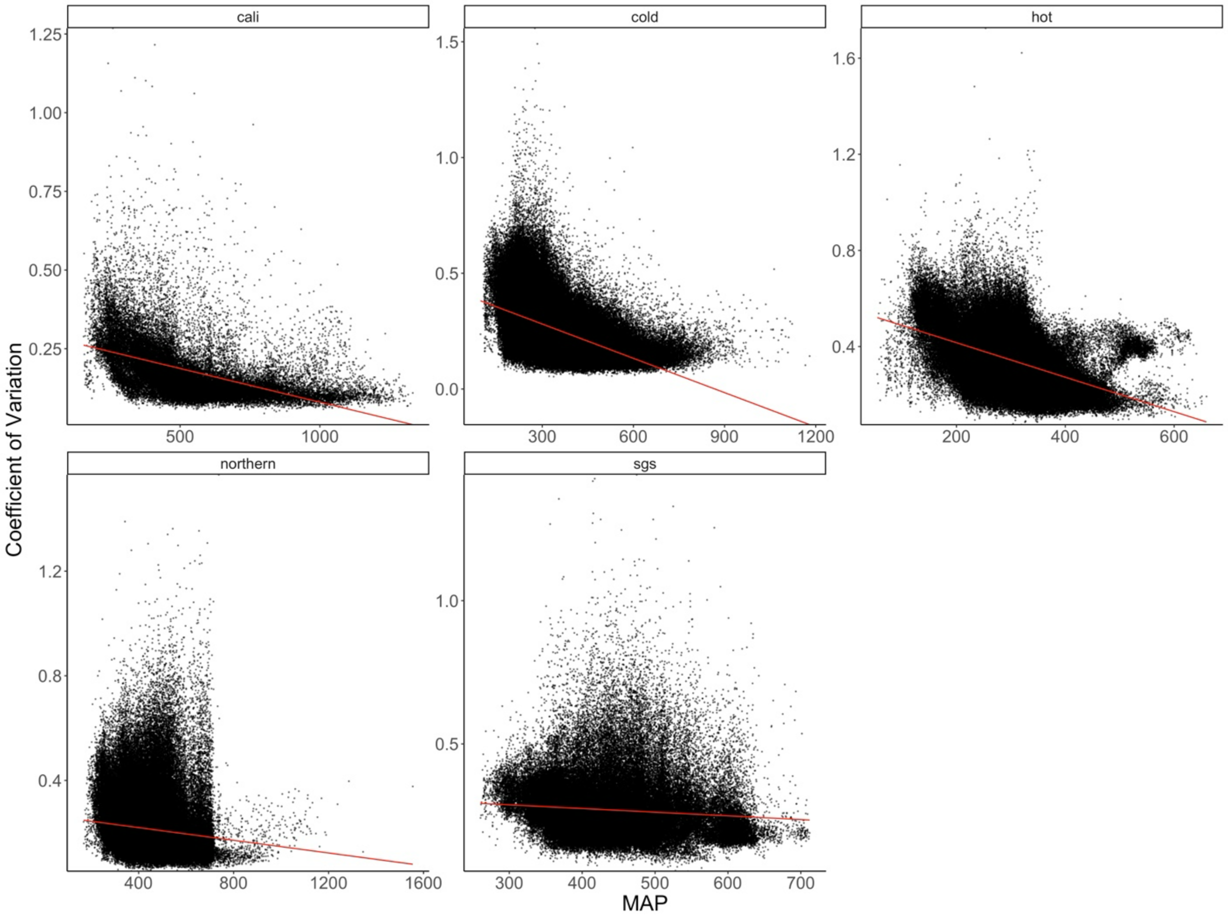

Figure S7. Relationship between MAP and coefficient of variation of NPP for each of the five ecoregions.

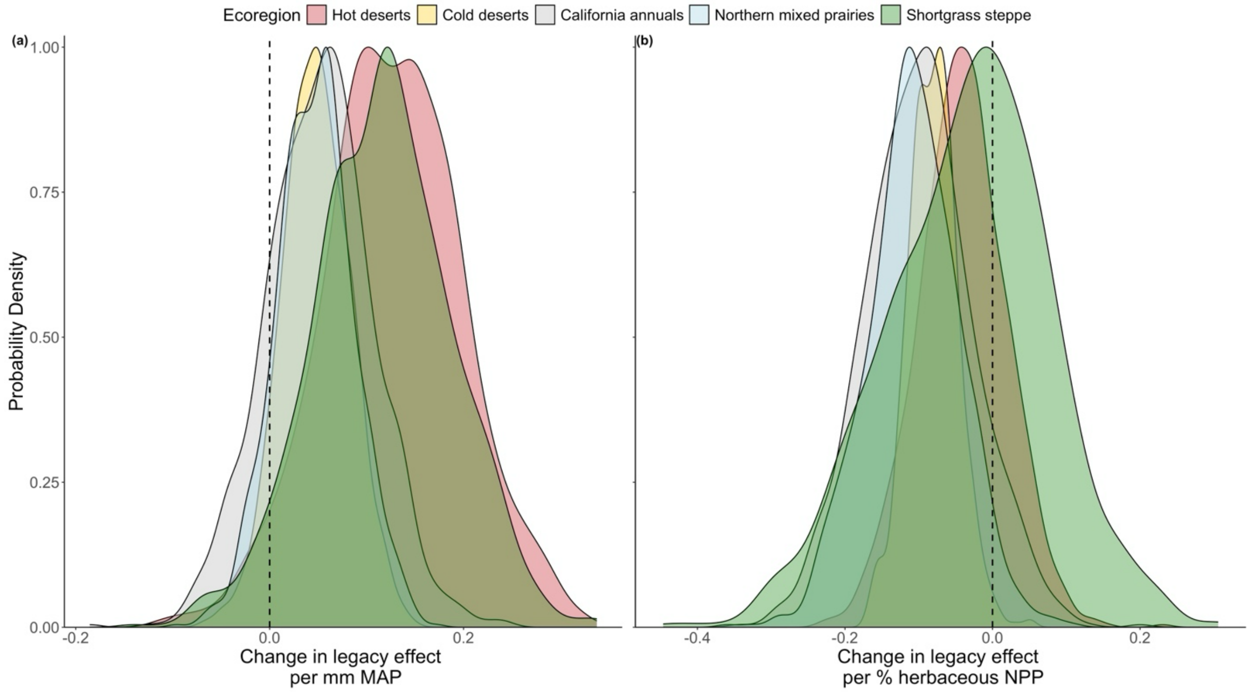

Figure S8. Probability density functions (scaled to 1) of **(a)** coefficients from the interaction between ecoregion, MAP, and previous-year NPP (Eq. 3) and **(b)** coefficients from the interaction between ecoregion, the fraction of herbaceous NPP, and previous-year NPP (Eq. 4) using standardized variables.

Methods S1 – Additional information on data processing

To analyze NPP in only rain-fed, herbaceous, grasslands we applied a series of steps to the NPP data for each ecoregion. First, the NPP data was cropped using shapefiles for each ecoregion. We then used USGS National Land Cover Database data (Dewitz 2021) to mask NPP pixels that were not shrub/scrub or grassland/herbaceous landcover types. This ensured that we were only analyzing herbaceously covered grasslands. NPP data was aggregated to a pixel resolution of 1500m to match the PPT data.

To further ensure that we were only using rain-fed, herbaceously covered grasslands in our analysis, we removed pixels based on five filtering steps. In order to ensure we were not using

unvegetated areas, we set a minimum boundary of productivity and removed any pixels that had a long term mean NPP below 10. We also removed any pixels that had a NPP value of zero for any year. To make sure we were only using rain fed areas, we calculated mean PPT use efficiency (PUE), the ration of average NPP to mean PPT. We removed any pixel that had a PUE value greater than or less than three standard deviations of the long term mean for each ecoregion, we also looked at PUE values in each year and removed pixels with anomalous PUE values based on the same three standard deviations. A high PUE value indicates irrigation, so removing the pixels with high values ensures that we are not using areas with irrigated agriculture in our analysis. Lastly, we calculated the slope of the ordinary least squares linear model of annual NPP responses to annual PPT values and removed any pixel with a slope greater than or less than three standard deviations of the mean, as a high slope would also indicate irrigation.

Analysis S1 – Determine the strongest 'legacy effect'

To assess the role of legacy effects on NPP, we first needed to select a lag variable to represent such effects. To this end, we consider multiple potential previous-year variables including previous-year NPP, PPT, or temperature to predict current-year NPP. In our models, lagged PPT, NPP, and temperature variables were calculated as the annual deviations from the long-term (36-year) mean to focus our analysis on interannual variability and to remove any effects of spatial gradients in these variables on NPP. Similarly, our response variable was also the NPP deviation from the long-term mean. However, a supporting analysis demonstrates that treating NPP as its original value or as a deviation from the long-term mean did not qualitatively change results. Nevertheless, for all analyses, we use deviations from the long-term mean for both the predictors and response variable (Analysis S1).

We first built three linear models to assess which lag variable was a stronger predictor of current-year NPP:

$$NPPdev_{x,t} = \alpha + \sigma lagPPT_{x} + \varepsilon_{x,t} \quad \text{(Eq. S1)}$$

$$NPPdev_{x,t} = \alpha + \sigma lagNPP_{x,t} + \varepsilon_{x,t} \quad \text{(Eq. S2)}$$

$$NPPdev_{x,t} = \alpha + \sigma lagTEMP_{x,t} + \varepsilon_{x,t} \quad \text{(Eq. S3)}$$

where $NPPdev_{x,t}$ denotes current-year NPP deviation from the long term mean at location x and time t, α denotes the intercept term, σ denotes a temporal slope that provides the relationship between current-year NPP deviation and the previous-year PPT deviation (Eq.S1) previous-year NPP deviation (Eq. S2) , or previous-year temp deviation (Eq. S3) ε denotes the error term.

This R^2 and AIC analysis determined that previous-year NPP deviation was a better predictor of current-year NPP deviations than both previous-year PPT deviation and previous-year temperature deviation. The model including previous-year NPP deviation as the predictor had a mean R^2 of .23, while the model including previous-year PPT had a mean R^2 of .0079 and the model including previous-year temperature had a mean R^2 of only .00094. Suggesting that previous-year NPP deviation can explain far more variation in current-year NPP than the other two previous-year variables (Fig. S1). AIC scores further confirmed this, as the model with previous-year NPP had

the lowest AIC score (Fig. S1). AIC scores and R^2 values from both model 1 (Eq. 1) and 3 (Eq 3). Were subtracted from model 2 (Eq. 2) for each iteration and 99% confidence intervals were calculated. 99% confidence intervals of mean R-squared differences and mean AIC differences between the models did not overlap with zero, indicating a significant difference between the models. Thus, in all subsequent analyses we used previous-year NPP deviation as our lag variable to quantify legacy effects on current-year NPP deviation.

Analysis S2

Additional analysis to determine differences in legacy effects due to vegetation functional group, particularly differences in legacy effects due to perennials versus annuals.

$$NPPdev_{x,t} = \alpha + \beta \mathrm{HERB} + \gamma lagNPP_{x,t} + \sigma lagNPP_{x,t}\ HERB \quad \text{(Eq. 6)}$$

$$NPPdev_{x,t} = \alpha + \beta AFG + \gamma lagNPP_{x,t} + \sigma lagNPP_{x,t} AFG \quad \text{(Eq. 7)}$$

$$NPPdev_{x,t} = \alpha + \beta \mathrm{PFG} + \gamma lagNPP_{x,t} + \sigma lagNPP_{x,t} PFG \quad \text{(Eq. 8)}$$

Where HERB, represent the proportion of NPP from herbaceous species, AFG represents the proportion of NPP from annual forbs and grass, PFG represents the proportion of NPP from perennial forbs and grass, α is the intercept term, β represents the main effect HERB (Eq. 6), AFG (Eq. 7) or PFG (Eq. 8), γ is the temporal slope giving the relationship between NPP and previous-year PPT at location x and time t, and σ is the spatiotemporal slope, giving the relationship between previous-year NPP and HERB (Eq. 6), AFG (Eq. 7), and PFG (Eq. 8)

Assessment of Temporal Autocorrelation

To assess the degree of temporal autocorrelation in our models, we first conducted a Durbin-Watson test on Eq.1, a model containing no lagged variables as predictors. To do this, we extracted Durbin-Watson values from each of the 1000 iterations of the model from the bootstrapping process and plotted the distribution of these values (**Fig S2)**. The mean Durbin Watson value was 1.96, which suggests weak positive autocorrelation (a value of 2 = no autocorrelation).

Eq. 2 served as a contrast in Eq. 1 in that is explicitly contained a lagged dependent variable (previous-year NPP). For this, we calculated the Durbin's h-statistic for Eq. 2, as this is more appropriate for a model with a lagged dependent variable as a predictor. Again, we calculated this value for all 1000 iterations of the model and extracted the values and plotted the distribution. The mean value was found to be 1.98 (**Fig S3)**. For Durbin's h-statistic a value of 1.96 indicates no autocorrelation, suggesting that our model has slight autocorrelation. We performed this same analysis on a subset of iterations for a more complex model (Eq. x) and arrived at the roughly the same test statistic. As such, we conclude that these data and our statistical models contain weak positive temporal correlation, but not of a sufficient magnitude to undermine their underlying inferences. Furthermore, the existence of positive autocorrelation is broadly consistent with results both from the models and from assessments of specific extreme-to-normal year transitions, and thus it is not surprising that our tests collectively indicate the presence of weak positive temporal autocorrelation.
